# Nutrition Literacy of Overweight/Obese and Non-Overweight/Obese Turkish Women and Affecting Factors

**DOI:** 10.1093/eurpub/ckac131.317

**Published:** 2022-10-25

**Authors:** EH Kozan Çıkırıkçı, MN Esin

**Affiliations:** 1 Nursing, Haliç University, Istanbul, Turkey; 2 Public Health Nursing, Istanbul University Cerrahpasa, Istanbul, Turkey

## Abstract

**Background:**

Nutrition literacy is having the skills and abilities required to prepare food, make healthy food preferences, and understand its effects on health, environment, and economy. Inadequate nutrition literacy can lead to an unhealthy diet, overweight, or obesity. Along with inadequate nutrition literacy, gender is also a risk factor for obesity. In this regard, it is seen that women’s level of nutrition literacy has an important role in gaining healthy eating habits and preventing chronic diseases related to nutrition such as obesity. This study aims to determine the level of nutrition literacy of adult Turkish women and the factors affecting.

**Methods:**

In this comparative descriptive research, 239 women were selected into 2 groups by their BMI, from Public Education Centers in Istanbul, Turkey, by using simple random sampling method. Data were collected using Introductory Characteristics Form and Adult Nutrition Literacy Assessment Tool. For statistical analysis, Pearson Chi-Square, Mann Whitney U and logistic regression were used.

**Results:**

In the study, adequate numerical literacy and food label reading rates in overweight/obese women were found lower (p = 0.000) than non-overweight/obese women. It was found that the number of main meals increases the nutrition literacy level of non-overweight/obese women 2.628 times (p = 0.012). In the overweight/obese group, it was found that number of children (p = 0.040), waist circumference (p = 0.048), snack amount (p = 0.022) and Youtube usage (p = 0.041) increase nutrition literacy levels.

**Conclusions:**

The nutrition literacy of both groups was found to be high. However it is highly affected by YouTube usage which provides a new perspective in terms of public health practices and policies. It is recommended for public health professionals to:

• use social media platforms and provide evidence-based information

• monitor the height, weight and BMI of individuals in the risk group

• organize interventions to strengthen nutritional literacy

**Key messages:**

• Adequate numerical literacy and food label reading sub-dimension mean scores of overweight/obese women was lower than the non-overweight/obese women.

• Spending time on Youtube increases the nutrition literacy levels in overweight/obese women by 91,116 times which points to the relationship between nutritional literacy and social media use.

